# Exploring university students’ nutrition literacy in Saudi Arabia: a cross-sectional survey

**DOI:** 10.3389/fnut.2024.1425650

**Published:** 2024-08-07

**Authors:** Najim Z. Alshahrani, Adeeb Ghazi Bafaraj, Hisham Mohammed Alamri

**Affiliations:** ^1^Department of Family and Community Medicine, Faculty of Medicine, University of Jeddah, Jeddah, Saudi Arabia; ^2^Faculty of Medicine, University of Jeddah, Jeddah, Saudi Arabia

**Keywords:** nutrition literacy, eating habits, factors, students, Saudi Arabia

## Abstract

**Introduction:**

Improving individuals’ nutrition literacy can be one way to promote healthy dietary behaviors, which lowers the incidence of non-communicable diseases. In Saudi Arabia, there is a limited information regarding nutrition literacy among students. Therefore, the purposes of this study was to investigate university students’ nutrition literacy and identify its predictors in Saudi Arabia. Another objective was to assess how nutrition literacy is associated with eating habits among this sample.

**Methods:**

This cross-sectional study was carried out among students attending the University of Jeddah and King Abdulaziz University (Jeddah City) in Saudi Arabia from June 2023 to September 2023. Using a convenience sampling approach, 570 students were enrolled in this study. An online-based structured survey tool including demographic details, a food frequency questionnaire, and a nutrition literacy scale was used to collect the data. Descriptive and multiple binary logistic regression analysis were performed.

**Results:**

Approximately 40.4% of the participants exhibited poor nutrition literacy. Female students were less likely than male students to have poor nutrition literacy (adjusted odds ratio, AOR = 0.62; 95% confidence interval, CI = 0.42, 0.92). Underweight (AOR = 1.73; 95%CI = 1.22, 4.56) and overweight participants (AOR = 3.1; 95%CI = 2.77, 7.23) were at higher risk of having poor level of nutrition literacy as compared to those who had normal weight. Students who did not take any nutrition-related courses had a 1.3 times higher probability of having a poor level of nutrition literacy compared to their counterparts (AOR = 1.3; 95%CI = 1.05, 3.74). Moreover, poor nutrition literacy was associated with higher weekly consumption frequencies of red meat, processed foods, funk foods and sugar-sweetened beverages.

**Conclusion:**

A higher percentage of university students had poor nutrition literacy, which was associated with gender, self-reported BMI status, nutrition-related courses and unhealthy eating habits. These findings can assist university administrators and policymakers in implementing effective measures to enhance students’ nutrition literacy rates in Saudi Arabia.

## 1 Introduction

A healthy diet is linked to better physical and mental health as well as quality of life. Nutritional status is a critical health indicator for determining a country’s health condition and morbidity profile (1). A poor diet is a key cause of chronic diseases such as diabetes, heart disease, stroke, cancer, and obesity, resulting in significant morbidity and untimely death (2). There are many factors that influence diet quality; nutrition knowledge is one of those which is often seen as a motivator for choosing a nutritious diet (3). Nutrition education is essential to manage the various aspects that impact individuals’ healthy eating habits and diet (4, 5). However, traditional nutrition education that focuses on knowledge has limited impact (6). In recent decades, nutrition literacy has emerged in nutrition education programs that encompass both the principles of nutrition knowledge and nutrition skills (6). Nutrition literacy is a subset of health literacy that focuses on how an individual can use nutrition-related health literacy to improve personal health in terms of eating habits (6). Nutrition literacy is defined in the literature as the ability of an individual to obtain, process, and understand nutrition information, as well as the skills required to make appropriate nutrition decisions (7, 8). Improving individuals’ nutrition literacy can be one way to promote healthy dietary behaviors (9), which lowers the incidence of non-communicable diseases (NCDs).

In Saudi Arabia, the prevalence of NCDs such as obesity, diabetes, cardiovascular diseases, etc. is rising quickly, posing a serious health concern for vulnerable groups (10, 11). For example, a recent survey including large sample of Saudi adults reported that the prevalence of overweight and obesity was 32.8 and 23.0%, respectively (12). According to a systematic review and meta-analysis (2000 to 2020 Yr.), the overall pooled prevalence of type 2 diabetes in Saudi Arabia was 16.4% (13). Evidence shows that NCDs are responsible for 73% of all mortality in Saudi Arabia (14), and also cause increased disease and financial burden. Irrespective of any age group (such as adolescents, adults, students, etc.), unhealthy and poor dietary habits are one of the predisposing factors of occurring NCDs and diet-related diseases (15–17). The role of nutrition literacy in the management, treatment, and prevention of NCDs is optimized (18). Furthermore, the burden of malnutrition is linked to low nutrition literacy, therefore, boosting nutrition literacy rates is an important part of encouraging healthy behaviors (19).

Earlier studies conducted across the world reported a range of nutrition literacy among different sub-populations. A recent study, for example, found a lower rate of adequate nutrition literacy (29%) among Palestinian adults (20). Other studies found that adolescents in Turkey (21) and adults in Bangladesh (22) have moderate nutrition literacy. Previous studies have shown that socio-demographic factors such as gender, occupation, family income, education level, maternal and paternal education level and place of residency are associated with nutrition literacy (20–23). Moreover, evidence shows that nutrition literacy was positively associated with healthy eating behaviors (24, 25).

Few studies in Saudi Arabia have examined nutrition literacy among adolescents and parents, revealing that a significant proportion had inadequate/poor nutrition literacy (34.9% to 46%) (23, 26, 27). A better understanding about the prevalence and factors of nutrition literacy among different vulnerable groups including university students is urgently needed to develop evidence-based and priority-wise initiatives and strategies. However, information regarding nutrition literacy among Saudi university students is relatively unknown. Generally, the university-aged period, which falls into an emerging adulthood period of lifespan, is often considered a key growing phase of life (28). University students go through this stage when many changes take place in their lives, including academic obligations, growing flexibility, and independence. All of these might make university students more vulnerable to adopting various unhealthy dietary practices and disordered eating (29, 30). This change mostly affects eating habits, unintentionally contributing to weight gain, which may have serious ramifications in the future, as weight increase in young adults’ period is regarded as a substantial risk factor for obesity in late adulthood. Therefore, this study was designed to bridge the knowledge and research gap by assessing nutrition literacy among university students in Saudi Arabia. The specific objectives of this study are as follows:

- To assess the prevalence of nutrition literacy among university students in Saudi Arabia.
- To explore the socio-demographic and other factors (body mass index, nutritional diseases, nutrition course, etc.) that are associated with students’ poor level of nutrition literacy.
- To investigate the association between students’ nutrition literacy and eating habits.

## 2 Materials and methods

### 2.1 Survey design and participants

Students from the University of Jeddah and King Abdulaziz University in Saudi Arabia participated in this cross-sectional analytical study. Both of these are public institutions located in the city of Jeddah (Makkah province). These universities are among the most prestigious educational and research institutions in the country, offering a wide range of degrees in disciplines like as medicine, engineering, science, education, etc.

The following selection criteria were used to recruit study participants: (i) being age ≥ 18 years (adults), (ii) being Saudi citizens and (iii) being currently enrolled students. Students suffering from fatal illnesses (such as diabetes, heart disease, etc.) and psychological disorders (like depression) were requested not to participate in this study in order to avoid the possibility of overestimation and/or underestimation of the findings. Furthermore, those who were not willing to participate were excluded from the study. The study was conducted between June 2023 and September 2023.

### 2.2 Sample size and sampling

The minimum number of participants required for this study was estimated using a single sample proportion formula with the following parameters: (i) a 44.6% prevalence of poor nutrition literacy was used (*p* = 0.45) based on the earlier data from Saudi adolescents (23) (since there was as no such data among university students in Saudi Arabia), (ii) 95% precision level (Z = 1.96), and (iii) 5% margin of error (d = 0.05). The formula is as follows:

Minimum sample size, $n=\frac{z^{2}\times p\times \left(1-p\right)}{d^{2}}=\frac{{\left(1.96\right)}^{2}\times 0.45\times \left(1-0.45\right)}{{\left(0.05\right)}^{2}}\;$ =380.3 ≈ 380.

A 15% non-response rate was also taken into account, yielding an ideal sample size of 437 (380 + 57). Finally, this study recruited a total of 570 students.

To recruit study participants, a convenient sample selection technique was used. An online version of the questionnaire was created using Google Forms, and the link was distributed to students at the selected universities (*n* = 2). The invitation to the survey was sent to the student’s institutional email accounts. The email body briefly stated the study’s objectives, eligibility requirements, and confidentiality declaration. Furthermore, the survey link was distributed to students attending those universities via personal communications of the data collectors via social media platforms such as WhatsApp. The online survey link was made available for 1 month to give students enough time to respond.

### 2.3 Contents of the questionnaire

A structured questionnaire with three sub-sections was used in this study. These include: (i) socio-demographic and health-related information, (ii) assessment of eating habits, and (iii) assessment of nutrition literacy. Various measures of the questionnaire were adapted by reviewing relevant literature (22, 24, 25, 31, 32). The questionnaire was finalized after conducting a pilot survey to ensure clarity of the items. Moreover, the content validity of the survey questions was determined by reviewing the items with academic experts.

The first part of the questionnaire was about sociodemographic and health-related information including gender, age, study subject, living area, family income, maternal education, parental education, self-reported BMI status, nutrition-related diseases, ever taken nutrition-related courses and self-perceived need for access to nutrition information. The status of BMI was assessed through a self-reported question: What is your BMI status? The possible options were underweight, normal weight or overweight.

In the next section, participants’ eating habits were assessed by a food frequency questionnaire (FFQ) (17, 31–33). This FFQ included 8 items with three possible options: “6–7 days a week,” “3–5 days a week,” and “<3 days a week.” Participants’ eating habits for fruits and vegetables, fish or sea foods, milk or dairy products, red meat, processed foods, junk foods, sugar-sweetened beverages, and chocolate/ice cream were included in this FFQ. Higher consumption frequency was regarded when consuming a food group for 5 to 7 days a week.

In the final section, participants’ nutrition literacy, the dependent variable of this study, was evaluated using a modified nutrition literacy scale established and validated by Liao et al. (24). The scale had 8 items under the following five domains of nutrition literacy: (i) obtain domain (2-items, evaluate respondents’ ability to search for, find, and acquire nutrition information), (ii) understand domain (2-items, assess the basic nutritional knowledge and abilities to grasp general nutrition information), (iii) analyze domain (one item, capacity to analyze nutritional information in a particular situation.), (iv) appraise domain (2-items, assess the capacity to judge and assess nutrition information in terms of personal needs), and (v) apply domain (one item, measures the capability application skill of nutrition information in daily life to achieve a healthy diet). The item number 4 was rephrased to maintain cultural integrity (such as “For me, being able to understand the contents of the food-based dietary guidelines for Saudi Arabia is …). Each participant’s response to the eight items was graded on a 4-point Likert-type scale ranging from 1 (extremely difficult) to 4 (very easy). The total nutrition literacy score for an individual was calculated by adding the number of responses. The total nutrition literacy score ranges from 8 to 32 with a higher score indicating higher nutrition literacy.

### 2.4 Statistical analysis

Data were coded and sorted into Microsoft Excel for quality checks before being imported into the statistical package for the social sciences (SPSS, version 23.0) for analysis. Reliability analysis was performed to check the internal consistency of the scales used in this study. The Cronbach’s alpha for the FFQ and nutrition literacy scale was 0.73 and 0.78, respectively, which indicates an acceptable internal consistency (34). As the nutrition literacy score was not normally distributed, the median value (median 22.0) was used as the cut-off point. Individuals with a score of 22.0 or more were considered to have adequate nutrition literacy, whereas those with a score of less than 22.0 were regarded to have poor nutrition literacy.

Descriptive statistics such as frequency, percentage, etc. were calculated. Chi-square function was applied to observe the distribution of nutrition literacy (adequate vs. poor) by participants’ sociodemographic and eating habits-related variables. Multiple adjusted binary logistic regression model was performed to assess sociodemographic and other predictors of poor nutrition literacy. Moreover, adjusted binary logistic regression models were fitted to observe how nutrition literacy was associated with higher frequency of a specific food consumption. All regression models were fitted based on the Hosmer and Lemeshow criteria. The statistical significance was assessed by *p*-values less than 0.05.

### 2.5 Ethics

The study protocol was approved by the Bioethics Committee of Scientific and Medical Research of University of Jeddah Registration Number HAP-02-J-094 (approval number: UJ-REC-181). All study procedures were carried out in accordance with relevant guidelines and regulations. Informed consent was obtained from all participants after explaining the objectives of the study. No remuneration was given to the participants and participation was entirely voluntary. Moreover, participants were assured that their personal data would be kept confidential.

## 3 Results

### 3.1 Participants’ characteristics

Of 570 participants, more than half of them (51. 6%) were female. The mean age of the participants was 22 years (SD: 3.70). More than one-third of the participants were studying in the engineering group (36.8%). Below 20 % of the participants reported themselves as underweight (16.0%) and overweight (18.6%). Nearly a quarter of the participants (22.3%) had taken nutrition-related courses. Participants’ socio-demographic profile is demonstrated in Table 1.

**Table 1:** Presentation of nutrition literacy status across participants’ background characteristics (*n* = 570).

Variable(s)	Total; *n* (%)	Nutrition literacy		*P*-value
		Adequate	Poor	
		340 (59.6)	230 (40.4)	
**Gender**				
Male	276 (48.4)	158 (57.2)	118 (42.8)	0.021^*^
Female	294 (51.6)	182 (61.9)	112 (38.1)	
**Age (in years)**				
18–21	146 (25.6)	89 (61.0)	57 (39.0)	0.132
22–25	309 (54.2)	188 (60.8)	121 (39.2)	
>25	115 (20.2)	63 (54.8)	52 (45.8)	
**Study subject**				
Medicine	80 (14.0)	57 (71.2)	23 (28.8)	0.177
Education	231 (40.5)	125 (54.1)	106 (45.9)	
Engineering	210 (36.8)	145 (69.0)	65 (31.0)	
Science	49 (8.6)	25 (51.0)	24 (49.0)	
**Living area**				
Own house	274 (48.1)	181 (66.1)	93 (33.9)	
Rental house	187 (32.8)	101 (54.0)	86 (46.0)	0.111
Dormitory	109 (19.1)	58 (53.2)	51 (46.8)	
**Family income (monthly, SAR)**				
< 5,000	93 (16.3)	54 (58.1)	39 (41.9)	
5,000–9,999	137 (24.0)	85 (62.0)	52 (38.0)	0.887
10,000–15,000	210 (36.8)	126 (60.0)	84 (40.0)	
> 15,000	130 (22.8)	75 (57.7)	55 (42.3)	
**Maternal education level**				
Illiterate	68 (11.9)	39 (57.4)	29 (42.6)	
Elementary school	99 (17.4)	67 (67.7)	32 (32.3)	0.046^*^
Intermediate school	140 (24.6)	59 (42.1)	81 (57.9)	
Secondary school	109 (19.1)	60 (55.0)	49 (45.0)	
University level	154 (27.0)	104 (67.5)	50 (32.5)	
**Paternal education level**				
Illiterate	40 (7.0)	22 (55.0)	18 (45.0)	
Elementary school	69 (12.1)	53 (76.8)	16 (23.2)	0.042^*^
Intermediate school	91 (16.0)	65 (71.4)	26 (28.6)	
Secondary school	115 (20.2)	56 (48.7)	59 (51.3)	
University level	255 (44.7)	144 (56.5)	111 (43.5)	
**Self-reported BMI**				
Under weight	91 (16.0)	43 (47.3)	48 (52.7)	
Normal weight	373 (65.4)	223 (59.8)	152 (40.5)	0.004^*^
Overweight	106 (18.6)	31 (29.2)	75 (70.8)	
**Nutrition-related disease**				
Yes	158 (27.7)	98 (62.0)	60 (38.0)	0.268
No	412 (72.3)	242 (58.7)	170 (41.3)	
**Ever taken nutrition-related course**				
Yes	127 (22.3)	81 (63.8)	46 (36.2)	0.002^*^
No	443 (77.7)	184 (41.4)	259 (58.5)	
**Self-perceived need for access to nutrition information**				
No need at al	27 (4.7)	18 (66.7)	9 (33.3)	
Somewhat of a need	99 (17.4)	55 (55.6)	44 (44.4)	0.072
Has a need	210 (36.8)	141 (67.1)	69 (32.9)	
Has a great need	234 (41.1)	126 (53.8)	108 (46.2)	

Italic and asterisk values indicate statistically significant (*p* < 0.05).

### 3.2 Status of nutrition literacy

In our sample, approximately, 40.4% of the university students had poor nutrition literacy. Table 2 summarizes the detailed information from participants’ replies to the nutrition literacy measure. Majority of the participants (77.9% to 80.4%) reported positive responses (easy to very easy) in the obtain domain of nutrition literacy scale (item 1 and 2). It implies that they did not find much difficulty to search for information about healthy-eating behaviors and nutrition-related issues. However, nearly two-thirds (60.7%) of the respondents reported they found a degree of difficulty in judging the appropriateness of the obtained information (Item 6). Moreover, more than half of the participants reported that they found some extent of difficulty in understanding the general dietary guidelines (50.5%) and choosing a methods for their health needs (52.1%) (item 4 and 7).

**Table 2:** Participants’ responses of nutrition literacy questionnaire (*n* = 570).

Questions	Extremely difficult (%)	Difficult (%)	Easy (%)	Very easy (%)
**Obtain domain**				
1.For me, when there are nutrition-related issues, knowing where to find the right information is	12 (2.1)	100 (17.5%)	364 (63.9%)	94 (16.5%)
2.For me, when I want to learn healthy-eating behaviors knowing where to find the right information is	9 (1.6%)	117 (20.5%)	340 (59.6%)	104 (18.3%)
**Understand domain**				
3.For me, being able to understand the contents of the Daily Food Guide is …	7 (1.2%)	121 (21.2%)	374 (65.6%)	68 (11.9%)
4.For me, being able to understand the contents of the Dietary Guidelines for Saudi Arabia is	42 (7.4%)	246 (43.2%)	208 (36.5%)	74 (13.0%)
**Analyze domain**				
5.For me, choosing foods from the nutritional point of view to distinguish food groups and functions is	13 (2.3%)	103 (18.1%)	315 (55.2%)	139 (24.4%)
**Appraise domain**				
6.For me, judging whether the nutrition information on the network is correct or not is	48 (8.4%)	298 (52.3%)	159 (27.9%)	65 (11.4%)
**Apply domain**				
7.For me, choosing a method that meets my health need when there are many recommendations for healthy diets is	31 (5.4%)	266 (46.7%)	228 (40.0%)	45 (7.9%)
8.For me, using the right nutrition information in daily life for healthy eating is	10 (1.8%)	154 (27.0%)	316 (55.4%)	90 (15.8%)

### 3.3 Predictors of poor nutrition literacy

Bivariate distribution and chi-square function showed that participants’ gender (*p* = 0.021), maternal education level (*p* = 0.046), paternal education (*p* = 0.042), self-reported BMI (*p* = 0.004) and nutrition-related courses (*p* = 0.002) were significantly correlated with nutrition literacy (Table 1).

Adjusted regression estimation showed that female students were less likely to have poor nutrition literacy compared to their male counterparts (OR = 0.62; 95%CI = 0.42, 0.92). Underweight (OR = 1.73; 95%CI = 1.22, 4.56) and overweight participants (OR = 3.1; 95%CI = 2.77, 7.23) were at higher risk of having poor level of nutrition literacy as compared to those who had normal weight. Students who did not take any nutrition-related courses had a 1.3 times higher probability of having a poor level of nutrition literacy compared to their counterparts (OR = 1.3; 95%CI = 1.05, 3.74) (Table 3).

**Table 3:** Adjusted regression model shows the sociodemographic and other factors associated with poor nutrition literacy among study participants.

Predictors	Adjusted regression model		*p*-value
	Odds ratio	95% CI	
**Gender**			
Male	Reference		
Female	0.62	0.42, 0.92	**0.018**
**Self-reported BMI status**			
Underweight	1.73	1.22, 4.56	**<0.001**
Normal weight	Reference		
Overweight	3.1	2.77, 7.23	**<0.001**
**Nutrition-related course**			
Yes	Reference		
No	1.3	1.05, 3.74	**0.007**

Only statistically significant results are shown in the table. The regression model included all background variables (11 variables, see Table 1). The adjusted regression model was fitted by Hosmer and Lemeshow test (Chi-square = 10.451 and *p*-value = 0.154). Bold values denote statistically significant.

### 3.4 Association between nutrition literacy and eating habits

Above one-third of the students (38.1%) reported that they consumed fruits and vegetables for <3 days a week. Approximately, two-thirds of the students (62.1%) consumed fish or sea foods for <3 days a week. Moreover, 26.0%, 24.9.0%, 25.1%, and 22.6% of participants had a habit of consuming red meat, processed foods, junk/fast foods, and drinking sugar-sweetened beverages for 6–7 days a week, respectively (Table 4).

**Table 4:** Distribution of nutrition literacy status based on participants’ self-reported eating habits (*n* = 570).

Variable(s)	Total; *n* (%)	Nutrition literacy		*p*-value
		Adequate	Poor	
**Fruits and vegetables**				
6–7 days a week	121 (21.2)	83 (68.6)	38 (31.4)	
3–5 days a week	232 (40.7)	145 (62.5)	87 (37.5)	0.003^*^
<3 days a week	217 (38.1)	78 (35.9)	139 (64.1)	
**Fish or seafood**				
6–7 days a week	64 (11.2)	42 (65.6)	22 (34.4)	
3–5 days a week	152 (26.7)	79 (51.9)	73 (48.0)	0.049^*^
<3 days a week	354 (62.1)	224 (63.3)	130 (36.7)	
**Milk or dairy products**				
6–7 days a week	98 (17.2)	48 (49.0)	50 (51.0)	
3–5 days a week	216 (37.9)	136 (63.0)	80 (37.0)	0.055
<3 days a week	256 (44.9)	156 (60.9)	100 (39.1)	
**Red meat**				
6–7 days a week	148 (26.0)	54 (30.0)	94 (70.0)	
3–5 days a week	217 (38.0)	87 (40.1)	130 (59.9)	0.008^*^
<3 days a week	205 (36.0)	136 (66.3)	69 (33.7)	
**Processed food**				
6–7 days a week	142 (24.9)	53 (37.3)	89 (62.7)	
3–5 days a week	235 (41.2)	101 (42.9)	134(57.1)	0.001^*^
<3 days a week	193 (33.9)	123 (63.7)	70 (36.3)	
**Junk/fast foods**				
6–7 days a week	143 (25.1)	63 (44.1)	80 (55.9)	
3–5 days a week	204 (35.8)	84 (41.2)	120 (58.8)	0.001^*^
<3 days a week	223 (39.1)	140 (62.8)	83 (37.2)	
**Sugar-sweetened beverage**				
6–7 days a week	129 (22.6)	52 (40.3)	77 (59.7)	
3–5 days a week	186 (32.6)	79 (42.5)	107 (57.5)	0.003^*^
<3 days a week	255 (44.7)	156 (61.2)	99 (38.8)	
**Chocolate/ice-cream**				
6–7 days a week	213 (37.4)	124 (58.2)	89 (41.8)	
3–5 days a week	136 (23.9)	86 (63.2)	50 (36.8)	
<3 days a week	221 (38.8)	130 (58.8)	91 (41.2)	0.611

Italic and asterisk values indicate statistically significant (*p* < 0.05).

The chi-square test shows that consuming fruits and vegetables (*p* = 0.003), eating fish or seafood (*p* = −0.049), eating red meat (*p* = 0.008), eating processed foods (*p* = 0.001), consuming junk/fast foods (*p* = 0.001), and drinking sugar-sweetened beverage (p = 0.003) were significantly associated with students’ nutrition literacy (Table 4).

Adjusted regression estimation has shown that participants with adequate nutrition literacy were more likely to consume a higher frequency of fruits and vegetables a week (OR = 1.37; 95% = 1.19, 2.94) compared to those with poor nutrition literacy. Participants with poor nutrition literacy were at higher risk of consuming higher frequency of red meat a week compared to their counterparts (OR = 3.19; 95%CI = 2.41, 5.18). Participants who had poor nutrition literacy had an increased probability of consuming a higher frequency of processed foods a week than their counterparts (OR = 2.29; 95%CI = 1.41, 4.12). A greater probability of consuming a higher frequency of junk foods a week was found among those had poor nutrition literacy (OR = 2.80; 95%CI = 1.70, 3.56), in contrast to those had adequate nutrition literacy. In addition, participants with poor nutrition literacy had three times more likely to consume a higher frequency of sugar-sweetened beverages a week compared to their fellow participants (OR = 3.43; 95CI = 2.72, 7.80) (Table 5).

**Table 5:** Binary logistic regression analysis shows how nutrition literacy predicts eating behaviors of study participants (higher consumption).

Independent variable(s)	Fruits and vegetables	Fish or seafood	Red meat	Processed foods	Junk Foods	Sugar-sweetened beverage
	OR [95%CI]	OR [95%CI]	OR [95%CI]	OR [95%CI]	OR [95%CI]	OR [95%CI]
**Nutrition literacy**						
Adequate	1.37 [1.19, 2.94]	0.92 [0.71, 1.32]	**-**	**-**	**-**	**-**
	*P* = **0.022**	*P* = 0.97				
Poor	RC.	RC.				
**Nutrition literacy**						
Adequate			RC.	RC.	RC.	RC.
Poor	**-**	**-**	3.19 [2.41, 5.18]	2.29 [1.41, 4.12]	2.80 [1.70, 3.56]	3.43 [2.72, 7.80]
			*P* = **0.008**	*P* = **0.003**	*P* = **0.012**	***P* = 0.002**

OR, odds ratio; CI, confidence interval; RC, reference category; and *p* = probability value. We performed six different regression models, considering six food groups as dependent variables. Each model was adjusted for participants’ age, gender, BMI and nutrition-related courses. Each adjusted regression model was fitted by the Hosmer and Lemeshow test. Bolded values represent statistically significant.

## 4 Discussion

This study investigated university students’ nutrition literacy in Saudi Arabia and identified that participants’ gender, self-reported BMI status and nutrition-related courses were associated with poor nutrition literacy. Moreover, poor nutrition literacy was associated with unhealthy eating habits (i.e., higher consumption frequency of red meat, processed foods, funk foods and sugar-sweetened beverages). The findings provide an evidence-based scenario for nutrition and health experts to plan and implement interventions to increase nutrition literacy and healthy eating habits among university students in Saudi Arabia, as this is one of the first studies in the country to look at nutrition literacy among this population group.

In this study, approximately four out of 10 university students had low nutrition literacy. This finding indicates a higher proportion of students were nutritionally illiterate, which is comparable to previous research (24). A study conducted by Liao et al. (24) reported that nutrition literacy was sub-optimal among Taiwanese university students. Another study from Iran reported that 50.9% of the participating students had borderline nutrition literacy (35). In contrast to our findings, an earlier study showed a higher percentage of adequate nutrition literacy among university students in the USA (i.e., 80.8% adequate nutrition literacy) (36). Disparities in nutrition literacy rates across studies may be attributable to demographic and regional differences, sample sizes, and assessment instruments employed to assess nutrition literacy. The high proportion of poor nutrition literacy in the present study suggests that nutrition literacy should be emphasized in the country’s nutrition policy schemes to promote nutrition literacy. A nationwide assessment is recommended to entirely comprehend the degree of nutrition literacy among university students in Saudi Arabia.

Specifically, our participants showed better capacity to search for and acquire information about healthy-eating behaviors and nutrition-related issues (i.e., obtain domain of nutrition literacy). This can be rationalized by the fact that most university students in Saudi Arabia have easy access to the Internet via their computers and smartphones, and continuously engaged in different social networking sites. In opposite to this domain, our findings demonstrated that university students felt difficulty in judging the appropriateness of the information (i.e., appraise domain). The similar finding was observed among college students in Taiwan (24). Sometimes students may unable to figure out an effective technique to evaluate the authenticity of Internet health resources or traditional resources (37). A prior study looked at online search tactics for health information among US college students and found that they were frequently perplexed about what makes accurate information (38). In general, there are numerous sources of nutrition information or knowledge; for example, social media and mass media are the primary sources of nutrition knowledge in Arab nations (39). Hence, strengthening university students’ ability to assess the accuracy of information is an imperative objective for nutrition literacy initiatives in Saudi Arabia.

Another concerning finding of our study is that university students mentioned they experienced some level of difficulty with grasping Saudi dietary guidelines. Dietary Guidelines for Saudis are provided and promoted; however, there is room for additional information dissemination and public engagement (40). Since the Dietary Guidelines are part of a comprehensive approach to preventing diet-related chronic diseases (such as cardiovascular disease, type 2 diabetes, obesity, etc.), the country’ health and nutrition departments should take priority-based necessary actions to implement and promote dietary guidelines among the mass population.

Our study demonstrated that female students had a lower probability of having poor nutrition literacy than male students. This finding is supported by previous studies conducted among different population groups (23, 41). The lower risk of poor nutrition literacy among females is justified by the evidence that female students in Saudi Arabia are more aware of food and nutrition than male students (29). Generally, females are more inclined than males in health care, diet, nutrition, and body weight, particularly throughout their university years (29, 41). Our findings suggest that university authorities should provide gender-based nutrition education interventions, especially targeting male students to increase their nutrition literacy.

The present study showed underweight and overweight participants were at greater risk of having poor nutrition literacy than those who had normal weight. A similar association was observed by Bookari (23), showing being overweight and underweight were connected with poor nutrition literacy. A study conducted by Bahramfard and colleagues in Iran found that students with normal BMI had higher average nutrition literacy (35). A logical explanation for this relationship is that overweight and underweight individuals may be unaware of basic nutrition knowledge, appropriate eating habits, and a health-promoting lifestyle, as well as susceptibility to eating disorders, which affects their nutrition literacy. However, there is limited research that assessing the relationship between nutrition literacy and BMI across the world. A recent study carried out among adolescents (14–19 years) in Lebanon found no significant correlation between different domains of nutrition literacy and abnormal BMI status (42). In general, BMI is a potential health indicator that is associated with multiple factors such as socio-demographics, dietary behaviors, lifestyle, etc., which may undermine the influence of nutrition literacy. Longitudinal studies are recommended to understand the association between nutrition literacy and BMI.

Another important finding of this study is that students who did not ever take any nutrition-related courses were more likely to have poor nutrition literacy than those who had taken nutrition-related courses. The aforementioned finding is supported by previous studies in a sample of adults and students (22, 43). A reasonable for this finding may be that individuals who have ever taken nutrition-related courses are better educated on optimum nutrition practices, which may have a significant impact on their health. Thus, university administrators should provide nutrition-related sessions, free online courses and nutrition education programs for their students so that they can gain a basic knowledge of food choices and nutrition, and improve their nutrition literacy. Further nutrition education intervention studies are recommended to improve peoples’ nutrition literacy, eating habits and health outcomes in Saudi Arabia.

When we analyzing the participants’ food consumption frequency, it has been shown that nearly 40 % of students (38.1%) consumed fruits and vegetables for <3 days a week. This result was corroborated by prior research, which found that students did not consume fruits and vegetables regularly or frequently (17). A recent study reported that only 4% Saudis consume five servings of fruit and vegetables a day (40). In addition, fish or sea foods consumption frequency was found to be low among study participants. According to a survey report, in Saudi Arabia, 55.3% of participants did not meet Dietary Guidelines recommendations for fish consumption. Thus, the policymakers and public health experts should take prompt actions regarding healthy food consumptions as a preventive measure for better health. Furthermore, approximately a quarter of the students in our sample consumed unhealthy foods such as red meat, processed foods, junk/fast foods, and sugar-sweetened beverages on 6–7 days per week. This finding is consistent with other Saudi Arabian studies, which found students’ most of the snacking patterns were unhealthy (31). These unhealthy eating habits have been identified as potential risk factors for obesity and cardiovascular disease (17, 33). Collectively, this finding highlights the need to develop and implement evidence-based and priority-based initiatives that promote the importance of proper nutrition and healthy eating habits among vulnerable groups such as university students in Saudi Arabia.

Our study also revealed that poor nutrition literacy was associated with unhealthy eating habits. In particular, poor nutrition literacy was associated with a higher frequency of red meat, processed foods, funk foods, and sugar-sweetened beverages consumption a week. Moreover, adequate nutrition literacy was associated with a higher frequency of fruit and vegetable consumption. The result is comparable to earlier studies conducted among adults and university students (7, 24, 25). Earlier research conducted among Taiwanese college students observed that higher levels of nutrition literacy and healthy eating behaviors are positively correlated (24). Another study conducted among Bangladeshi adults found nutrition literacy was associated with healthy dietary habits (25). However, Natour et al. (20) found a modest association between nutrition literacy and eating behavior among Palestinians. This association sensitizes university authorities to design nutrition educational programs so that students can adopt healthy dietary habits and food preferences. Because nutrition literacy is a changeable predictor of food consumption pattern, it may have a favorable impact on the need for public counseling and awareness-raising initiatives to promote health and well-being.

### 4.1 Policy implications

The Saudi Food and Drug Authority (SFDA) and university authorities can take the output of this study to develop a nutrition intervention program for improving nutrition literacy levels among students across the country. The findings support policymakers with guidance on what factors to consider when designing interventions. Moreover, policymakers can use this study’s findings to develop new policies or modify existing policies regarding nutrition that support the prevention of nutrition-related risk factors for NCDs such as obesity. This study’s outcome can be used by university authorities to develop institution-based healthy eating habits programs or nutrition education for their students that promote how they get, process, and understand basic food and nutrition-related information.

### 4.2 Limitations

This study had certain drawbacks. First, the cross-sectional design of this study precludes causal relationships. Second, since the study was limited to two universities in the city of Jeddah, Saudi Arabia, the results cannot be generalized across the country. Third, the external validity of research findings may be limited due to the use of a non-probability sampling technique (i.e., sampling biases may present). Fourth, there was the possibility of respondents’ social desirability and reporting bias. Finally, because participants’ BMI status was not determined by anthropometric measures of height and weight, self-reporting bias may have existed in the study.

## 5 Conclusion

A higher percentage of university students had poor nutrition literacy, which was associated with gender, self-reported BMI status, nutrition-related courses and unhealthy eating habits. These findings may be important considerations for university administrators and policymakers in Saudi Arabia when implementing successful approaches to improve students’ nutrition literacy rates. Given Saudi Arabia’s commitment to “Health in All Policies” and “Vision 2030,” which include the up-gradation of the healthcare system, these findings contribute to the country’s disease prevention efforts and call for the promotion of healthy eating habits. Further longitudinal studies that include larger samples are recommended to identify factors of nutrition literacy, which could potentially assist in developing nutrition education interventions to improve nutrition literacy in Saudi Arabia.

## Data availability statement

The original contributions presented in the study are included in the article/supplementary material, further inquiries can be directed to the corresponding author.

## Ethics statement

The study protocol was approved by the Bioethics Committee of Scientific and Medical Research of University of Jeddah Registration Number HAP-02-J-094 (approval number: UJ-REC-181). Informed consent was obtained from all participants after explaining the objectives of the study.

## Author contributions

NZA: Conceptualization, Data curation, Formal analysis, Funding acquisition, Investigation, Methodology, Project administration, Resources, Software, Supervision, Validation, Visualization, Writing – original draft, Writing – review & editing. AGB: Data curation, Funding acquisition, Investigation, Methodology, Writing – original draft, Writing – review & editing. HMA: Funding acquisition, Investigation, Methodology, Resources, Writing – original draft, Writing – review & editing.
